# Are patients picking up what we are putting down? Considering nocebo effects in exercise for musculoskeletal pain

**DOI:** 10.3389/fpsyg.2023.1291770

**Published:** 2023-11-23

**Authors:** Ben Cormack, Giacomo Rossettini

**Affiliations:** ^1^Cor-Kinetic, London, United Kingdom; ^2^School of Physiotherapy, University of Verona, Verona, Italy

**Keywords:** nocebo effects, expectation, pain, exercise, musculoskeletal, physical activity, strength and conditioning, running

## 1 Introduction

Clinicians can encounter many difficulties in motivating their patients to participate actively in the rehabilitation process and encouraging active coping strategies. This can result in a high risks of treatment failure (McLeod et al., 2023), with phrases such as, “*How difficult is it to get patients to do exercises at home?*”, “*How difficult is it to get patients to do physical activity?*”, being frequently uttered by clinicians who find themselves managing patients with musculoskeletal (MSK) pain.

Recently, the scientific community has identified nocebo effects as negatively influencing therapeutic outcomes in MSK pain, and this suggests the importance of considering nocebo in both the clinic and in research (Hohenschurz-Schmidt et al., 2022). Nocebo effects (Latin “I shall harm”) represent the effects of the negative psychosocial context surrounding the application of different treatments, including exercise (Rossettini et al., 2022). For example, nocebo effects could be the result of perceived negative therapeutic acts induced by clinician's inappropriate words or behaviors during an exercise. These nocebo effects can influence the patient's expectations, leading to changes in both the mind and body, and in outcomes such as pain (Petersen et al., 2014). Despite the high negative value of nocebo effects, they are still poorly understood or considered by clinicians managing MSK pain (Palese et al., 2019).

Patients' expectations (e.g., of recovery outcome) have previously been shown to be a prognostic factor in MSK pain, with a positive expectation indicating a greater probability of recovering from painful problems (Hayden et al., 2019). Nocebo effects could potentially negatively influence recovery expectations in several ways, with regards to exercise, from perception of treatment suitability to negative beliefs surrounding the bodies capabilities (Rossettini et al., 2022). Avoiding nocebo effects is one part of reshaping patient beliefs to align with best evidence-based practice around treatments such as exercise (Benz and Flynn, 2013). These nocebo effects could be behind some of the problems clinicians frequently encounter, particularly managing the therapeutic exercise process.

Exercise and physical activity are core treatments for MSK pain in many international treatment guidelines (Bernstein et al., 2017), and although clinicians perceive that they have a powerful medicine (exercise) at their disposal, they can also fail to make patients perceive it as such (Segar et al., 2016). Various studies describe the barriers capable of limiting engagement with physical activity and exercise in the MSK fields (Jack et al., 2010). Perceived barriers, such as a lack of time, often limit participation and helping patients overcome these maybe be a key to increasing the effectiveness of exercise-based interventions (Jack et al., 2010) and reducing negative perceptions of recovery. An analogy could be like going to a Michelin-starred restaurant and being served a delicious dish but served up in a way that means it is perceived as low quality fast-food: it loses value, is no longer enjoyed and (in the worst case) even discourages patients from participating, through the creation of these nocebic effects.

In this Opinion paper, we first discuss the idea that a proportion of the potential ineffectiveness of exercise interventions may stem from issues related to the nocebo effects and subsequent recovery expectations. Then, we propose suggestions to mitigate nocebo effects during exercise prescription and application. Finally, we suggest the next steps, calling to action clinicians and researchers in the MSK field to recognize the potential negative influence of nocebo effects in exercise.

## 2 Discussion

### 2.1 Nocebo effects and exercise: pitfalls

#### 2.1.1 Are clinicians speaking the same language with their patients?

One proposed source of nocebo effects is the divergence in communication that may exist during the prescription and application of exercise. When the two protagonists in the therapeutic encounter, the patient and the clinician are not aligned, for example, on the type of exercise to be performed (e.g., yoga vs. weight training) or on the mode of execution (e.g., in the form of play and recreational activity vs. with barbells), there is a risk of creating nocebo effects that may lead to negative expectations, lack of engagement, tensions, rupture of the therapeutic alliance and drop-out from the rehabilitation pathway (Miciak and Rossettini, 2022). Scenarios such as these can occur when the relationship between patient and clinician is not harmonious, leading the two protagonists to speak different “languages” (e.g., the patient wants to voice concerns and worries, while the clinician is interested in exercise form or biomechanics rationale) (Miciak and Rossettini, 2022). On the one hand, clinicians are inclined to offer a scientific message that exercise represents the best evidence-based treatment; on the other hand, it is expected that this translates into observable behavioral changes (e.g., the transition from physical inactivity to more active styles) (Hansford et al., 2022). The focus from clinicians can often be on communicating the number of repetitions or sets to be performed, the amount and intensity of loads to be used, and the frequency and duration of the activity to be performed without fully considering whether these messages are relevant to patients' perspectives or goals (O'Keeffe et al., 2016).

The question is: *Are clinicians sure that their messages are understood in the patients' language?* Sometimes, the best evidence-based message is not the best message for the patients, and this can potentially cause harm and potentially alter expectations of outcomes.

#### 2.1.2 What kind of exercise experience are clinicians offering their patients?

Another potential source of nocebo effects we propose is the exercise experience that clinicians offer their patients with MSK pain. A suboptimal rehabilitation experience, this can encompasses affective, motivational and psychological components, could potentially negatively influence the patient's willingness to perform an exercise or even an entire exercise program (Rossettini et al., 2022). Two clinician-related aspects may be considered here: their explicit information conveyed, and also their overall behavior. These two aspects, including their manner, personal beliefs and behaviors, can be considered separate but also connected (Cook et al., 2023). Potentially, clinicians may instill negative beliefs and expectations by providing exercise explanations based on biomechanical models that could lead to avoidance instead of action (e.g., back flexion to be avoided in lifting) (Bunzli et al., 2017). For other patients, their perspectives, preferences, desire's and expectations regarding exercise are not considered (Rossettini et al., 2022) resulting in boring, uninspiring and demotivating proposals that alienate the patient from the perception that physical activity can be enjoyable and fun. There is a potential for clinicians' advice, however well-meaning, to negatively influence rehabilitation. This could be through avoidance of activities or specific positions (e.g., avoiding movements of the head in case of neck pain, or creating fear around activities or specific positions (e.g., reducing running in case of low back pain) (Rossettini et al., 2022). This could lead to the overall therapeutic benefit of exercise being reduced and negatively alter recovery perceptions. Identifying those with negative expectations of recovery could also aid recovery and an opportunity to focus on trying to change them (DiSanti et al., 2018).

The question is: *Are clinicians aware that their thoughts, beliefs, and actions are correctly perceived by their patients?* Sometimes, clinicians' mindset could lead us to be more part of the problem than part of the solution.

### 2.2 Nocebo effects and exercise: opportunities

**Figure 1:**
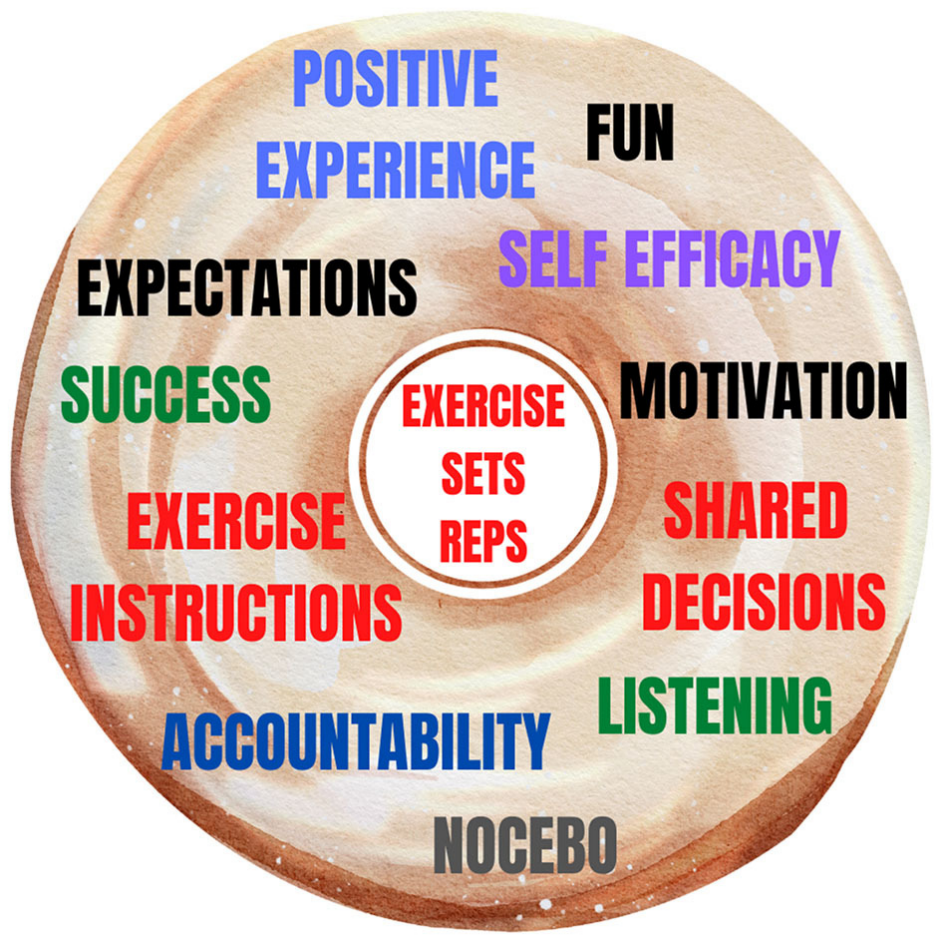
The “exercise donut” to avoid nocebo effects. The “exercise donut” could be a useful way to conceptualize some of the elements that clinicians and researchers could consider to make exercise more effective in terms of both practical implementation and therapeutic effects and avoiding nocebo effects. The focus with exercise can often fall on the exercise itself and the performance of the exercise rather than the person exercising. This focus on the exercise is conceptualized as the hole of the donut, the whole of the donut should also be considered to avoid nocebo effects and maximize positive effects through interaction and practical application.

In Figure 1, we report the “exercise donut” to summarize the variables that clinicians can use to mitigate nocebo effects.

#### 2.2.1 The need of nurturing a positive relationship in exercise

As the therapeutic relationship between clinician and patient can generate nocebo effects in MSK pain, nurturing the therapeutic relationship is crucial to mitigate them (Blasini et al., 2018). A positive therapeutic relationship also can maintain patient engagement to an exercise program and generate positive expectations of outcome, therefore, clinicians should be aware of their verbal and non-verbal interactions (Babatunde et al., 2017a), equally a poor relationship could harm outcome expectancies. Exercise prescription should involve person-centered interactions and shared decision-making, instead of just focusing on specific adaptations in the MSK system (Powell et al., 2023). Effective listening also contributes toward building a strong therapeutic alliance and avoiding therapeutic rupture (Babatunde et al., 2017b). A key element in involving patients in the exercise process is understanding patient perspectives, expectations, and preferences (Vader et al., 2021). The communication around exercise engagement can often focus on external motivators, with messaging such as “exercise is medicine” (Sallis, 2009). Potential intrinsic motivators that are extracted from patient interactions and narratives could also be used to enhance engagement and create a link between exercise and patient derived goals (Gardner et al., 2019). Prescribing exercise parameters that align with goals and communicating this alignment of goal and exercise prescription could enhance the intrinsic motivation that has been shown to improve performance and exercise adherence (Teixeira et al., 2012).

#### 2.2.2 The need of creating a positive experience with exercise

Negative therapeutic experiences that MSK pain patients experience can induce nocebo effects, negative outcome expectations and emotional responses such as fear of reinjury. Hope and fear are key part of outcome expectations (Carroll et al., 2016) and a positive experience should involve positive messages and emotions rather than nocebic elements. To avoid these nocebo effects and potentially enhance the therapeutic effects of exercise, clinicians should listen to patients' stories and analyze their previous experiences and use this to plan future exercise programs. Moreover, focusing on parameters that create a positive emotional experience could become a focus of clinicians (Wienke and Jekauc, 2016). Movement and exercise afford the opportunity to embody and contextualize the positive messages that the body is strong and robust (Cormack et al., 2023). Exercise feedback is often based around potentially negative emotional elements such as incorrect exercise form and harmful outcomes, and positive motivational messaging may increase the positive affect and maintain the analgesic effects of exercise (Vaegter et al., 2020). Over-medicalization and focus on technicalities within therapeutic exercise may lead to less focus placed on their person and their emotional state (Podlog et al., 2014); the emotional state may play a role in the participation and subsequent perceived outcomes of exercise-based interventions.

### 2.3 Nocebo effects and exercise: next steps

The optimal dosage (e.g., sets and repetitions) and type of exercise (e.g., aerobic, strength, and conditioning) for many MSK complaints have yet to be identified, and this continues to represent a conundrum for clinicians (Cashin et al., 2022). While this evidence may be disconcerting, it allows MSK clinicians and researchers to think outside the box, considering nocebo effects as active phenomena capable of influencing the prescription and application of exercise. In Table 1, we synthetized implications for MSK clinicians and researchers managing exercise.

**Table 1:** Implications for musculoskeletal clinicians and researchers managing exercise.

**Stakeholders**	**Approach**
Clinicians	• Consider the messaging around exercise, not just the exercise itself; • Use the patients' language during exercise prescription and administration; • Involve patients in the decision-making process around exercise; • Create positive exercise experiences that could enhance the effects of the exercise; • Consider patients' values, beliefs, expectations, preferences and previous experiences when setting up the exercise; • Build and nurture a strong therapeutic relationship with your patients.
Researchers	• Investigate the patient's perception of negative elements influencing the prescription and application of the exercise; • Consider the barriers the clinician perceives during exercise prescription and administration with the patient; • Tests the efficacy of different relational and communicative styles during the exercise prescription and application; • Assesses the temporal impact (e.g., short, medium, and long term) of nocebo effects during the exercise prescription and application; • Assesses the impact in different outcomes (e.g., pain and disability) and health care costs (e.g., patient, NHS) of nocebo effects during the exercise prescription and application; • Studies the prognostic role of expectations, beliefs, mindset and previous negative experiences in inducing nocebo effects during the exercise prescription and application.

#### 2.3.1 Implications for clinical practice and research

From a clinical perspective, there is a strong need to raise awareness and training among MSK pain clinicians about the role of nocebo effects in exercise by providing them strategies that can be used in rehabilitation (Hohenschurz-Schmidt et al., 2022) that set patients up for success with exercise experiences that create “small wins” and may avoid nocebo effects through failure or the need for excessive coaching from clinicians. This may involve tasks that are within the capabilities of the patient's skill or symptom tolerance, and the positive psychological state generated maybe used to progress the challenge of an exercise or exercise program. Communication around exercise needs to focus not only on the language of the clinician, but also on the patient. A hyper-focus on exercise form could be more damaging than helpful if not balanced with positive feedback. Creating a strong narrative between the exercise program and a positive outcome might also improve patient perception of exercise as a suitable treatment for their problem and improve overall outcome perceptions. When selecting an exercise, clinicians should align with patient values and preferences, potentially enhancing intrinsic motivation and participation in the exercise program. Clinicians should focus on building a therapeutic relationship with patients as a precursor to exercise interventions involving their patient in the decision-making processes. Finally, creating a positive experience through feedback and success may help avoid nocebo effects and even enhance the effects of exercise.

Future research is needed to understand the impact of nocebo effects in MSK exercise prescription and application in different fields (e.g., prevention and rehabilitation), considering the status of the participants (e.g., sportsmen and sedentary), their conditions (e.g., healthy and patients) and the neurophysiological mechanisms of pain (e.g., nociceptive, neuropathic, and central sensitization). For example, quantitative (e.g., surveys) and qualitative studies (e.g., focus groups and semi-structured interviews) should investigate patients' and clinicians' perceptions of the elements around exercise prescription and application that potentially induce nocebo effects. Furthermore, future randomized controlled trials should evaluate the efficacy on outcomes such as pain and disability of the same therapeutic exercise (e.g., strength and conditioning training) offered with different styles of therapeutic relationship between clinician and patient (e.g., empathetic style with respect to marketing interactions).

## 3 Conclusion

Nocebo effects are real and may influence the prescription, practical application, and perceived recovery outcomes from exercise in MSK pain. In this opinion paper, we highlight the importance of considering nocebo effects in clinical and research contexts to manage the complexity of patients with MSK pain. We call on clinicians and researchers to consider not just the exercises that they are using in clinical practice but the context into which they are applied and the effect on expectations they could generate. This context, involving the emotion, relationship, and messaging around exercise, may “make or break” how effective the exercise prescription becomes and should be food for thought for all those in MSK care.

## Author contributions

GR: Conceptualization, Supervision, Visualization, Writing – original draft, Writing – review & editing. BC: Conceptualization, Writing – original draft, Writing – review & editing.
